# Evidence of gender bias in True-False-Abstain medical examinations

**DOI:** 10.1186/1472-6920-9-32

**Published:** 2009-06-07

**Authors:** Shona Kelly, Reg Dennick

**Affiliations:** 1 Division of Epidemiology and Public Health, University of Nottingham, Nottingham UK; 2 The Medical School, University of Nottingham, Nottingham, UK

## Abstract

**Background:**

There is evidence that males and females differ in their attainment on a variety of assessments in general and in medical education. It has been suggested that the True-False-Abstain (TFA) format with negative marking is biased against females.

**Methods:**

Eight years worth of examination data from the first two years of an undergraduate medical curriculum was analysed. 359 courses were evaluated for statistically significant differences between the genders using ANOVA. Logistic regression was used to test if subject area, calendar year or exam format predicted that males or females do better (termed male advantage or female advantage).

**Results:**

Statistically significant differences between the genders were found in 111 (31%) of assessments with females doing better than males in 85 and males better in 26. Female advantage was associated with a particular year (2001), the Personal and Professional Development strand of the curriculum, in course assessment and short answer questions. Male advantage was associated with the anatomy and physiology strand of the curriculum and examinations containing TFA formats, where the largest gender difference was noted. Males were 16.7 times more likely than females to do better on an assessment if it had any questions using the TFA format.

**Conclusion:**

Although a range of statistically significant gender differences was found, they were concentrated in TFA and short answer formats. The largest effect was for TFA formats where males were much more likely to do better than females. The gender bias of TFA assessments in medical education is yet another reason why caution should be exercised in their use.

## Background

Assessment is a key component of teaching and its effective use in medical education can assist in meeting curriculum goals and maintaining standards acceptable to the profession and to the public [1]. It is acknowledged that assessment should be fair and defensible, reliable and valid and that it should promote the deep learning of appropriate domains of knowledge, skills and attitudes. In addition it should accommodate individual differences and, by using a wide range of formats, should not disadvantage any particular group of learners

In terms of individual differences a recent trend for females to out perform males in schools and universities has added to the dispute on gender differences between male and female minds with a recent debate at Harvard garnering press and online coverage [1]. But the issue is contentious and evidence that supports or refutes differences can be mustered by either side. Explanations for differences have involved gender preference for type of question format, differences in innate skills, and a tendency by females to avoid taking risks in comparison to males [2,3].

Gender preferences for particular types of assessment have produced some considerable debate [2], but little empirical research in the educational literature. Most of the work has been carried out on children and adolescents and much less exists on university students. Some have suggested that females do better on in-class assessments as opposed to unseen exams [2] but a test of this at the University of Sussex found that females did better than males in both formats over a wide range of coursework [3].

A large study looking at the topic of MCQs and gender bias was conducted by the Educational Testing Service in the USA [4] involving millions of students ranging from 9 year olds to graduate school students, including those taking the MCAT (Medical College Admissions Test). Asking students to construct the answer rather than select the answer did not generate gender bias when the same question was asked in different formats. This has also been shown by other authors [5-7]. However, answers requiring written responses favoured females and those requiring the production of a figure or the interpretation of graphical information favoured males.

A range of studies have looked at gender issues in medical education. Female medical students have been shown to do better than males in Objective Structured Clinical Examinations (OSCEs) and other types of clinically based performance examinations [8-10]. In a meta-analysis Ferguson et al showed that females do better than men in clinical training and assessment and are more likely to obtain an honours degree[11]. Females are also more likely to obtain honours in the graduate-entry medical course (BMBS degree) [12]. Furthermore female gender has been shown to be a positive predictor of clinical reasoning in a graduate entry PBL course [13].

However, one particular category of assessment instrument has been identified as allegedly generating negative female bias, namely the True-False-Abstain (TFA) format of examination questions. The advantages of the TFA format are that large numbers of examinees can be tested with relatively few resources, that marking is objective, that large areas of knowledge as well as specialist, in depth topics can be covered, that poor non-discriminatory questions can easily be identified and that large question banks are available. Nevertheless it has been found that there were significant gender effects when true/false questions were used in maths exams (Anderson 1989 as cited in [14]) and these differences were attributed to a female tendency to avoid risk-taking (Forgasz 1991 as cited in [14]). In older mathematics students the female superiority was restricted to specific types of mathematical knowledge [15]. Research also suggests that gender differences from the MCQ format may only occur in the students with the highest ability [5] which are the group of students most likely to be enrolled in medical school. Problems with the TFA format have led the University of Western Australia, Faculty of Medicine and Dentistry to ban their use on the grounds that the format "lends itself to guessing, testing trivial knowledge and the promotion of rote learning." [16].

Research on the evidence for the impact of negative marking summarised on the Higher Education Academy's Medicine, Dentistry and Veterinary Medicine website in the UK [17] concluded that there is a weak systematic gender bias with males scoring higher than females. However most of the work reported was conducted on high school students in the US who are younger than the students in our medical schools and who have a wider range of scholastic abilities.

In summary, the literature suggests that there are real gender differences in student's performance in assessments and that these differences may be attributed to the format of the assessment. In particular it has been suggested that TFA formats may disadvantage females. In order to test these assertions we decided to analyse 8 years worth of exam data for gender bias prior to instituting a new modular programme and assessment scheme as part of developments on our medical curriculum. A significant portion of these exams consisted of TFA questions with negative marking.

## Methods

The data was provided by the Faculty Office of the Medical School and consisted of all final official individual-level course scores and course descriptions in the first two years of the undergraduate medical programme between 1995, when exam results became easily available in electronic form and 2002, prior to significant modifications to our curriculum. This period of 16-student years was characterised by a continuity of curriculum content and assessment methods. For each exam there is also information on:

1) the subject area/content: Theme A: molecular sciences (the cell), Theme B: anatomy & physiology (the man), Theme C: non-medical aspects of health (the society), Theme D: personal & professional development (the doctor), and

2) the number and format of questions asked.

A variable to assess the format of exam questions indicated whether the exam contained any of each of the following formats; course work, essay, in-class assessment, lab studies, OSCE, short answer, single phrase, spotter, single word answer, true/false abstain questions, or Viva. Other available information used was gender, age and overall mark for the year.

All TFA exams were machine read using Multiquest OMR exam papers and the raw TFA scores were obtained from the appropriate Multiquest data files.

All analyses were conducted using SPSS v. 11.5 for Windows. Within each year ANOVA (analysis of variance) was used to identify statistically significant differences between the mean male and female score for each course (approximately 45 comparisons) and thus the unit of analysis was the course. All analyses were corrected for multiple comparisons (i.e. p < 0.011). A data file was then created that indicated, for each course within each year, the presence of a statistically significant gender difference, the magnitude of the difference, the subject area/content and the format of the exam questions as indicated above.

Logistic regression was used to test whether the subject area/content, calendar year or each exam format, individually, predicted that males or females do better (termed, male advantage or female advantage). Logistic regression is a statistical model where the outcome variable is dichotomous. In this case two outcomes were assessed: 1) females mean scores for an exam were statistically greater than male's – 'female advantage' and, 2) males mean scores for an exam were statistically greater than female's – 'male advantage'. Variables significant in univariate logistic regression analysis were then entered into a multivariate logistic regression to predict the two outcomes.

To examine the proportion of right and wrong answers and abstentions generated by males and females the raw marks for the top 15 TFA examinations showing bias against females was analysed and the overall proportion of right, wrong and abstain scores calculated.

## Results

### Description of the data

There was data available from 359 course offerings. Statistically significant differences between the genders, after correcting for multiple comparisons, were found in 111 (31%) of assessments. Overall females did better than males in 85 (24% of all assessments and 77% of the assessments with a gender difference).

Essay-type questions were the only form of assessment in 50 (14%) assessments, true/false questions only in 88 (24%) and in-class assessments only in 57 (16%). Table 1 describes the breakdown by assessment type – numbers add up to more than 100% as 13% of classes use multiple forms of assessment. Eighty-seven percent of exams used only one assessment format, 11% used two and 1% use three. As one might expect OSCE, and Viva formats were used alone. Essays, short answer questions, single phrase and true/false questions were the formats most likely to be combined in assessments.

**Table 1:** Most frequent assessment formats

**assessment format**	**proportion of all assessments**
course work	8.6%
Essay	15.3%
in class assessment	5.3%
lab studies	1.9%
OSCE	4.4%
short answer	25.8%
single phrase	5.0%
spotter	3.9%
single word answer	2.2%
true/false/abstain	36.9%
Viva	1.4%

Most courses had at least one year between 1995 and 2002 with significant gender differences (data not shown). On average 1/3 of the courses (see Table 2) in any given year show a statistically significant difference in the marks between the genders with 2000 showing the smallest proportion (26%) and 2001 the largest proportion (55%).

**Table 2:** Gender differences by calendar year

	1995	1996	1997	1998	1999	2000	2001	2002
total number of themes	46	46	46	46	46	44	43	43
number where females excel	13	5	11	14	6	10	20	6
number where males excel	2	5	2	2	8	1	0	6
% where females excel	28%	11%	24%	30%	13%	23%	47%	14%
% where males excel	4%	11%	4%	4%	17%	2%	0%	14%

### Univariate predictors of gender differences in mean course scores

Univariate logistic regression was used to examine the effect of each variable, individually, on the outcomes and the results are listed in Table 3. For 'female advantage' there was a significant positive association for the calendar year 2001, Theme D (the Doctor) vs. all others, having some in-class assessments, and having some short answer questions. The variables that show a statistically negative association with 'females-do-better' are Themes A (the cell), B (the man) and C (the society) vs. all others, and having some T/F questions on the assessment. The largest odds ratios are seen with only T/F questions and having some in-class assessment.

**Table 3:** Variables that were significantly associated with female advantage or male advantage in univariate analyses

**Variable**	**B**	**SE**	p *	**OR**
**Females advantage**				

Year				
1995			not sig	
1996			not sig	
1997			not sig	
1998			not sig	
1999			not sig	
2000			not sig	
2001	1.773	.536	0.001	5.89
2002	reference			
Theme A vs. all others	-.823	.312	.008	0.44
Theme B vs. all others	-.816	.260	.002	0.44
Theme C vs. all others	-.823	.312	.008	0.44
Theme D vs. all others	1.498	.322	<.001	4.47
having some in-class assessment	1.822	.493	<.001	6.19
having some short-answer questions	1.008	.264	<.001	2.74
having some TFA questions	-1.700	.345	<.001	0.18
				

**Males advantage**				

Theme A vs all others	-1.248	.624	.045	0.29
Theme B vs all others	2.315	.622	<.001	10.12
Theme C vs all others	-1.248	.624	.045	0.29
having some short answer questions	-1.548	.745	.038	0.21
having some TFA questions	3.260	.744	<.001	26.04

corrected for multiple comparisons

OR = Odds ratio; Theme A = the cell; Theme B = the man; Theme C = the society; Theme D = the doctor.

The variables with a statistically significant positive association with 'male advantage' (see Table 3) are Theme B (the man), and having some T/F questions. While a statistically significant negative association is seen with Themes A (the cell) and C (the Society) vs. all others, and having some short answer questions. The odds ratios for having some T/F questions are extremely large!

### Multivariate analysis for female advantage over males

In multivariate analysis for 'female advantage' in-class assessment was no longer a significant predictor when theme and calendar year were entered into the equation. In other words the difference in course marks, seen between males and females, in courses assessed by in-class assessment were 'explained' by the theme and calendar year rather than by the fact that the course was assessed in-class. The final model showed that females were 5.9 times as likely to do better than males in the calendar year 2001, and only 26% and 13% likely to do better than males in Themes B (the man) and C (the society). They were only 18% as likely to do better than males when the exam contained at least some T/F questions (Table 4). The apparent advantage that females have in exams with short answer questions is extremely small (odds ratio (OR) = 1.03, p < .001) and not likely to account for the superior performance of females overall in medical school.

**Table 4:** Multivariate Logistic Regression – final model for female advantage

**variable**	**B**	**SE**	**p**	**OR**
Year = 2001	1.769	.413	<.001	5.87
Theme B vs all others	-1.355	.344	<.001	0.26
Theme C vs all others	-2.025	.398	<.001	0.13
having some TFA questions	-1.718	.398	<.001	0.18
having some short answer questions	1.394	.317	<.001	1.03
Constant	-.308	.245	.209	0.74

OR = Odds ratio; Theme B = the man; Theme C = the society;.

### Multivariate analysis for male advantage over females

Few variables explained why males do better than females on exams (Table 5). Only Theme B (the man) (4.6 times the odds of doing better) and having some T/F questions explained the difference. If at least some T/F questions were on the exam males were 16.7 times more likely to score higher than females.

**Table 5:** Multivariate Logistic Regression – final model for male advantage

**Variable**	**B**	**SE**	**p**	**OR**
Theme B vs all others	3.260	.744	.017	4.64
having some TFA questions	2.816	.757	<.001	16.71
constant	-5.515	.841	<.001	.004

OR = Odds ratio

### Explanations for gender differences

It has been suggested that the reason why females perform less well in TFA examinations is due to their increased abstention rates in comparison to males who are less likely to abstain. This hypothesis is supported by the following results. Using the combined raw data of correct answers, wrong answers and abstentions from a sample of the top 15 TFA exams showing large gender differences, it was calculated that there is a 3% difference in abstaining between males and females (females abstaining more than males, p < .002) and a corresponding 3% difference in correct answers between males and females (males greater than females, p < .008). The proportion of wrong answers is not significantly different between the genders implying that differences in correct answers are due directly to differences in abstaining behaviour. However, the data do not allow one to distinguish between increased abstaining by females or decreased abstaining by males.

## Discussion

This paper makes a contribution to the controversy about gender differences in assessment performance and the possibility that differences can be explained by the format of examination questions. We found that males were vastly more likely than females to do well on an assessment when the exam contained some true/false questions (OR = 16.71, p < .001) and when the content of the assessment was anatomy and physiology (Theme B; the man) (OR = 4.64, p <.017). In contrast female advantage was in one calendar year (OR = 5.87, p < .001) and when the assessment contained some short answer questions. The apparent advantage that females have in exams with short answer questions is extremely small (OR = 1.03, p < .001) and not likely to account for the superior performance of females overall in medical school.

The interesting finding in this project is the extreme gender differences in the effect of TFA questions on generating statistically significant differences in final assessment marks between the genders. We were able to show that the difference was not completely due to course content although there was some suggestion of a cohort effect as female students in the calendar year 2001 were more likely to do better than their male counterparts in that year.

This result adds to the growing number of disadvantages that have been attributed to TFA exam formats. For example they are considered to test only a limited range of cognitive skills mainly aimed at the remembering and understanding level at the lower end of Bloom's Taxonomy [18]. This means that TFA exams tend to be aimed at factual recall and hence they encourage the rote and surface learning of factual information [19,20]. Good practice suggests that overall curriculum strategy should encourage assessments that make learners learn in useful and relevant ways [21]. In addition it has been pointed out that TFA questions cannot discover if a student correctly identifying a false statement actually knows the correct answer [20,22]. There is also ambiguity in the wording of many TFA questions. This problem in question construction was exposed by the work of Holsgrove & Elzubeir [23] who examined a series of MB BS finals papers and membership. Case and Swanson, who wrote the seminal work on constructing objective tests in medical education [24] do not recommend the use of TFA questions and the National Board of Medical Examiners in the USA has stopped using this format. In the UK no Royal College is now using TFA tests for membership.

From a theoretical perspective the negative marking of TFA questions affects their validity [25]. TFA questions are assumed to validly assess items of factual knowledge but the addition of negative marking introduces two other abilities into the assessment. Firstly the student has to think about their confidence with the answer and secondly they have to make a judgment, based on a variety of other factors that may include willingness for risk-taking ability, previous experience, etc. [26]. Thus the validity of TFA questions is compromised by these two additional factors and the student's score can be more influenced by their exam technique and risk taking behaviour rather than their knowledge [27]. It can also be argued that negative marking is unethical. Students have marks they have legitimately acquired from a correct answer taken away from them because they gave an incorrect answer to an unrelated question.

To the above disadvantages must now be added the evidence that TFA questions are gender biased: males doing better than females. The original rationale for the introduction of negative marking and the option of abstaining with TFA questions was that it was supposed to encourage students to be honest about their understanding and to discourage guessing. This was supposed to produce a more 'professional' attitude towards knowledge which modelled its use in the clinical setting. However, it is debatable whether this 'professional' argument is now relevant. In the UK the Royal Colleges have abandoned TFA formats for professional clinical exams and replaced them with extended matching formats. The gender data reported here do not support this 'professional' argument and even suggest that the TFA format gives a guessing *advantage *to males [4,17]. The explanation for this phenomenon is not clear but the common argument used is that it is associated with the greater risk taking behaviour of males. However, it could be equally due to the more cautious behaviour of females or different problem solving strategies.

Nevertheless, it could be argued that the reason males outperform females on TFA formats is that they know more and simply give more correct answers. There is no a priori reason why this should be the case and the fact that they average the same number of wrong answers implies similar levels of knowledge. The simplest explanations are that females abstain more than males or that males guess more than females. Thus the difference is caused by the format of the exam interacting with gender differences rather than its content.

This study has the advantage of systematically collected data in large datasets collected over multiple cohorts of students. The disadvantage is the lack of other potentially explanatory variables. We have begun a new project to examine the role of other potential explanatory factors for these gender differences.

## Conclusion

The implications of this result cast further doubts over the validity of TFA assessments and provides further evidence that this format of examination should be treated with caution. Medical schools still using this type of examination should evaluate their examination data for evidence of gender bias.

## Competing interests

The authors declare that they have no competing interests.

## Authors' contributions

RD initially identified the potential problem, SJK did the data analysis, both authors wrote the paper.

## Pre-publication history

The pre-publication history for this paper can be accessed here:


